# Precipitate obtained following membrane separation of hydrothermally pretreated rice straw liquid revealed by 2D NMR to have high lignin content

**DOI:** 10.1186/s13068-015-0273-4

**Published:** 2015-06-18

**Authors:** Kengo Sasaki, Mami Okamoto, Tomokazu Shirai, Yota Tsuge, Hiroshi Teramura, Daisuke Sasaki, Hideo Kawaguchi, Tomohisa Hasunuma, Chiaki Ogino, Fumio Matsuda, Jun Kikuchi, Akihiko Kondo

**Affiliations:** Organization of Advanced Science and Technology, Kobe University, 1-1 Rokkodaicho, Nada-ku, Kobe, Hyogo 657-8501 Japan; RIKEN Biomass Engineering Program, 1-7-22 Suehiro-cho, Tsurumi-ku, Yokohama, Kanagawa 230-0045 Japan; Department of Chemical Science and Engineering, Graduate School of Engineering, Kobe University, 1-1 Rokkodaicho, Nada-ku, Kobe, Hyogo 657-8501 Japan; Department of Bioinformatic Engineering, Graduate School of Information Science and Technology, Osaka University, 1-5 Yamadaoka, Suita, Osaka 565-0871 Japan; RIKEN Center for Sustainable Resource Science, 1-7-22 Suehiro-cho, Tsurumi-ku, Yokohama, Kanagawa 230-0045 Japan

**Keywords:** Rice straw, Hydrothermal pretreatment, Lignin, Nanofiltration, Enzymatic hydrolysis, Black precipitate

## Abstract

**Background:**

Hydrothermal pretreatment of lignocellulosic biomass such as rice straw can dissolve part of the lignin and hemicellulose into a liquid fraction, thus facilitating enzyme accessibility to cellulose in bioethanol production process. Lignin is awaited to be recovered after hydrothermal pretreatment for utilization as value-added chemical, and lignin recovery also means removal of fermentation inhibitors. To recover lignin with high content from the liquid fraction, it is necessary to separate lignin and hemicellulose-derived polysaccharide. Therefore, the following processes were applied: membrane separation with nanofiltration (NF) and enzymatic hydrolysis by hemicellulase. To clarify lignin-concentrated fraction obtained during these processes, the fates of lignin and polysaccharide components were pursued by a solution NMR method and confirmed by compositional analysis of each fraction.

**Results:**

After hydrothermal pretreatment of rice straw, the NF concentrate of the supernatant of liquid fraction was hydrolyzed by hemicellulase and the resulting black precipitate was recovered. In this black precipitate, the intensity of NMR spectra related to lignin aromatic regions increased and those related to polysaccharides decreased, compared to rice straw, the solid fraction after hydrothermal pretreatment, and the NF concentrate. The lignin content of the black precipitate was 65.8 %. Lignin in the black precipitate included 52.9 % of the acid-insoluble lignin and 19.4 % of the soluble lignin in the NF concentrate of supernatant of liquid fraction.

**Conclusion:**

A precipitate with high lignin content was obtained from supernatants of the liquid fraction. These results suggested that precipitation of lignin was enhanced from concentrated mixtures of lignin and hemicellulosic polysaccharides by hydrolyzing the polysaccharides. Precipitation of lignin can contribute to lignin recovery from lignocellulosic biomass and, at the same time, allow more efficient ethanol production in the subsequent fermentation process.

**Electronic supplementary material:**

The online version of this article (doi:10.1186/s13068-015-0273-4) contains supplementary material, which is available to authorized users.

## Background

Lignin is a heterogeneous aromatic biopolymer that is the second most abundant polymer after cellulose on earth [1, 2]. Lignin valorization, converting lignin to higher value compounds, is one of the greatest challenges for biorefineries [3]. Currently, utilization of cellulose and hemicellulose in lignocellulosic biomass such as agricultural materials, softwood, and hardwood has been a challenge in production of bioethanol for use as a transportation fuel with low greenhouse gas emissions [4, 5]. First, pretreatment of biomass is necessary to break down the structure of lignin and facilitate enzyme access to carbohydrates [6–8]. Therefore, technology to utilize lignocellulosic biomass, including pretreatment, enzymatic hydrolysis, and fermentation by specialized organisms, has been developed [9–11]. In this process, the remaining lignin in lignocellulosic biomass should be recovered [12], to allow utilization of lignin as a high-value-added product in such forms as polymers and carbon fibers [13]. Recovery of lignin also means removal of fermentation inhibitors, because high concentrations of lignin reduce bioethanol production [14].

One of the most abundant types of lignocellulosic biomass, rice straw [15], has been utilized for bioethanol production. A hydrothermal pretreatment has been used because this process is cost-saving and environmentally benign due to no catalyst requirement and low corrosion [7]. Hydrothermal pretreatment of rice straw produces a hemicellulose-rich liquid fraction and cellulose-rich solid fraction [16]. Lignin is distributed in both the liquid and solid fractions [14]. Membrane separation, a reduced-energy process, can recover lignin and hemicellulose and remove fermentation inhibitors such as weak acids and furan derivatives that are produced in hydrothermal pretreatment [17, 18]. Recently, ultrafiltration (UF) membranes were utilized to recover lignin of 1000–2000 Da from kraft cooking liquor [18, 19]. However, lignin was intricately mixed with polysaccharides after the hydrothermal pretreatment [14]. Separation of lignin from polysaccharides is desirable to recover purified lignin.

Our previous research [14] utilized a nanofiltration (NF) membrane, which has a smaller pore size than UF membrane [18], UF membrane, and enzymatic hydrolysis by hemicellulase, on a liquid fraction of hydrothermally pretreated rice straw. This process increased ethanol production from hemicellulosic sugars by removing fermentation inhibitors [14]. This process can also be optimized for lignin recovery. A solution-state two-dimensional (2D) ^1^H-^13^C heteronuclear single quantum coherence (HSQC) nuclear magnetic resonance (NMR) method can resolve lignin and polysaccharide components in depth in plant cell wall materials [20, 21]. Solution-state NMR has advantages of high resolution and comparative quantification of numerous sample components [22, 23]. Therefore, solution-state NMR analysis can pursue the fate of lignin and polysaccharide components during above membrane separation and enzymatic hydrolysis on liquid fraction of hydrothermally pretreated rice straw. Thus, this method can identify the fraction containing high levels of lignin.

The aim of this study was to obtain the fraction containing high lignin content from the liquid fraction of hydrothermally pretreated rice straw, using solution-state 2D NMR analysis. Lignin was highly concentrated in a black precipitate that appeared after enzymatic hydrolysis of NF concentrate.

## Results and discussion

### Membrane separation of liquid fraction of hydrothermally pretreated rice straw

**Fig. 1:**
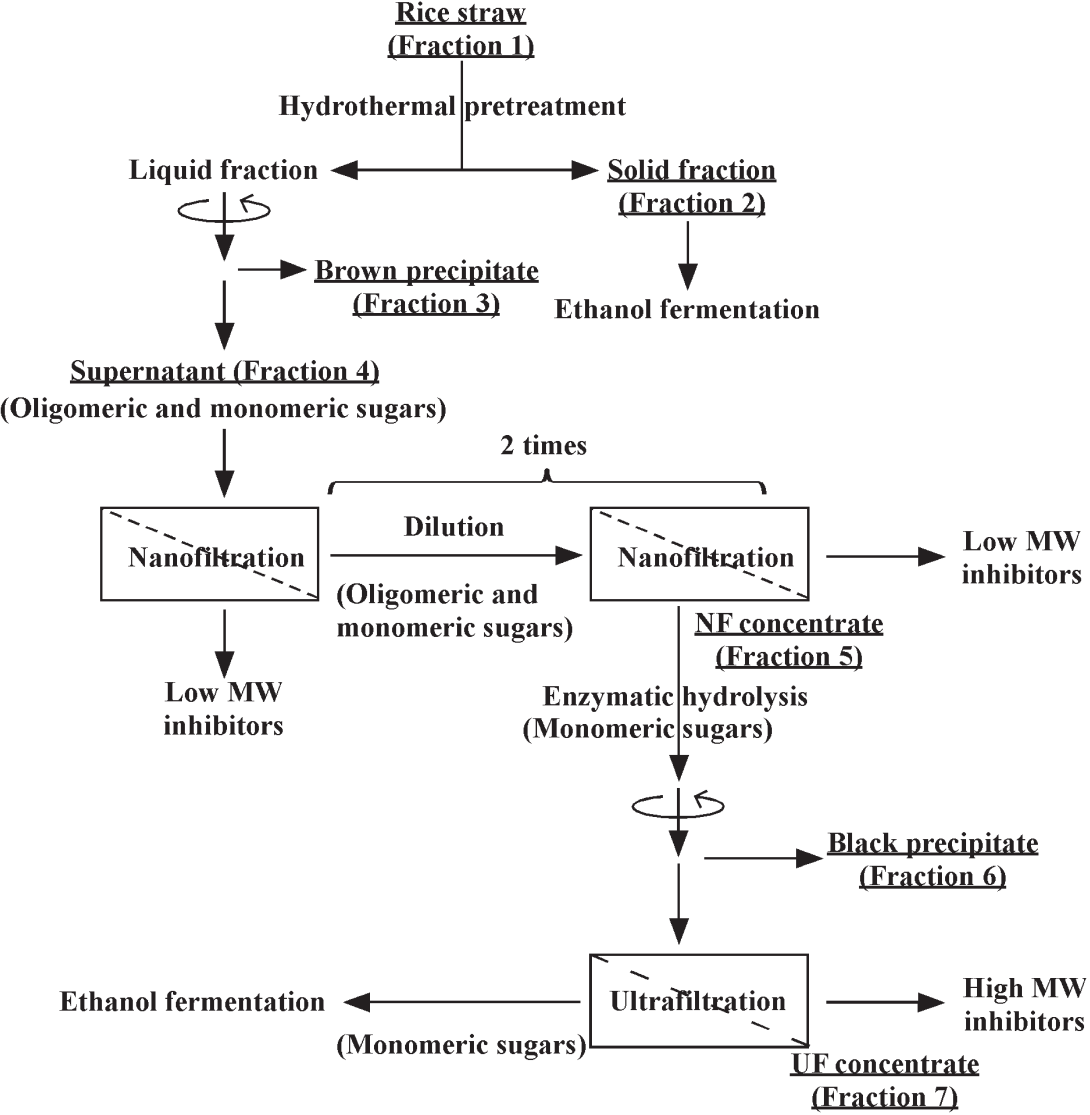
Schematic flow for ethanol fermentation from hydrothermally pretreated rice straw. Hydrothermal pretreatment of rice straw gives the solid and liquid fractions. The supernatant obtained after centrifugation of the liquid fraction, which contains oligomeric and monomeric sugars, was concentrated by nanofiltration (NF), followed by two rounds of dilution and NF concentration, to remove low molecular weight (MW) (<150 Da) fermentation inhibitors. Then, oligomeric sugars in the NF concentrate were enzymatically hydrolyzed to monomeric sugars, and the resulting black precipitate was recovered. Finally, the fraction with monomeric sugars was permeated through an ultrafiltration (UF) membrane to remove high MW (≥150,000 Da) fermentation inhibitors. This UF permeate and solid fraction can be fermented by *Saccharomyces cerevisiae* for ethanol production [14]

In a previous study, a membrane separation process was optimized to recover hemicellulosic sugars from the liquid fraction of hydrothermally pretreated rice straw for subsequent ethanol fermentation [14]. In current research, seven fractions were sampled to clarify the fates of lignin and polysaccharide (Fig. 1). Rice straw (fraction 1) and the solid fraction of hydrothermally pretreated rice straw (fraction 2) were sampled. The brown precipitate obtained after centrifugation (fraction 3) was sampled, as was the resulting supernatant (fraction 4). The supernatant (fraction 4) was concentrated by NF, then diluted and concentrated by NF twice (fraction 5) to remove low molecular weight (<150 Da) fermentation inhibitors [14]. This NF concentrate (fraction 5) was enzymatically hydrolyzed by hemicellulase, and the black precipitate appeared (fraction 6). The supernatant of the enzymatically hydrolyzed NF concentrate was filtered through UF to remove high molecular weight (≥150,000 Da) fermentation inhibitors. This UF concentrate was sampled (fraction 7). The components related to lignin and polysaccharides of these fractions were analyzed in detail following 2D NMR.

### Fates of lignin and polysaccharide components revealed by 2D NMR

**Fig. 2:**
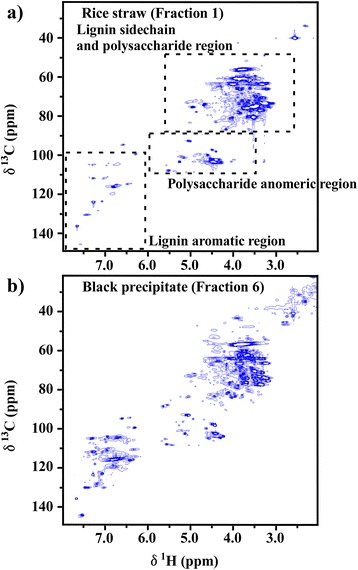
2D NMR spectra of **a** rice straw (*Fraction 1*) and **b** black precipitate (*Fraction 6*). Details of Fractions are presented in Fig. 1

2D NMR followed lignin and polysaccharide components in rice straw (fraction 1), the solid fraction (fraction 2), brown precipitate (fraction 3), NF concentrate (fraction 5), black precipitate after enzymatic hydrolysis (fraction 6), and UF concentrate (fraction 7) (Fig. 1). 2D NMR spectra were obtained (Fig. 2 and Additional file 1). The assigned NMR data are summarized in Tables 1 and 2.

**Table 1:** Chemical shifts (δ_13C_/δ_1H_) (ppm) of lignin components assigned in this study (DMSO-*d*_6_ and pyridine-*d*_5_ = 4:1)

		α(7)	β(8)	γ(9)	2	3	5	6
Aromatic region	Syringyl				104.5/7.03, 104.5/6.94, 104.5/6.84			104.5/7.03, 104.5/6.94, 104.5/6.84
	Guaiacyl				111.7/7.21		116.0/7.17, 116.1/7.08, 115.1/7.02, 114.9/6.88, 115.0/6.79	116.0/7.17, 116.1/7.08, 115.1/7.02, 114.9/6.88, 115.0/6.79
							119.5/7.06, 119.5/6.97, 119.1/6.88	119.5/7.06, 119.5/6.97, 119.1/6.88
	*p*-Hydroxyphenyl				128.3/7.30, 129.8/7.33			128.3/7.30, 129.8/7.33
	Ferulate		111.6/7.42, 115.7/6.45					123.6/7.22
	*p*-Coumarate	145.4/7.73			130.5/7.66, 130.3/7.60, 130.3/7.54, 130.3/7.48	116.2/6.95, 114.5/6.64, 114.1/6.51, 114.0/6.41	116.2/6.95, 114.5/6.64, 114.1/6.51, 114.0/6.41	130.5/7.66, 130.3/7.60, 130.3/7.54, 130.3/7.48
Aliphatic side chain	Cinnamyl alcohol			61.9/4.26				
	β-O-4	73.0/5.16, 72.2/5.10, 72.3/5.04						
	β-O-4-H/G (*erythro*)	71.2/4.97	84.9/4.44	60.8/3.44				
	β-O-4-H/G (*threo*)		84.1/4.52	60.7/3.82				
	β-O-4-S		86.7/4.25					
	β-5	87.4/5.64	52.2/3.73					
	5-5/4-O-β	83.2/5.11	87.7/3.62					

Methoxyl groups were assigned at 56.1/3.92 and 56.0/3.77 ppm

**Table 2:** Chemical shifts (δ_13C_/δ_1H_) (ppm) of polysaccharide components assigned in this study (DMSO-*d*_6_ and pyridine-*d*_5_ = 4:1)

	1	2	3	5
(1,4)-β-D-Glc*p*	103.2/4.57, 103.3/4.36			
(1,4)-β-D-Glc*p*(R)	97.2/4.58			
(1,4)-α-D-Glc*p*(R)	92.7/5.17			
2-*O*-Ac-β-D-Xyl*p*	99.7/4.68	73.8/4.74		
3-*O*-Ac-β-D-Xyl*p*	101.9/4.62		75.3/5.03	
(1,4)-β-D-Xyl*p*	102.0/4.46			63.5/3.46, 63.4/4.15, 63.7/4.07, 63.3/3.35
(1,4)-β-D-Xyl*p*(R)	98.0/4.48			
(1,4)-α-D-Xyl*p*(R)	92.8/5.08			
α-L-Ara*f*	107.8/5.63			
α-L-Fuc*p*	100.6/5.34, 101.1/5.26			

### Lignin aromatic regions

**Fig. 3:**
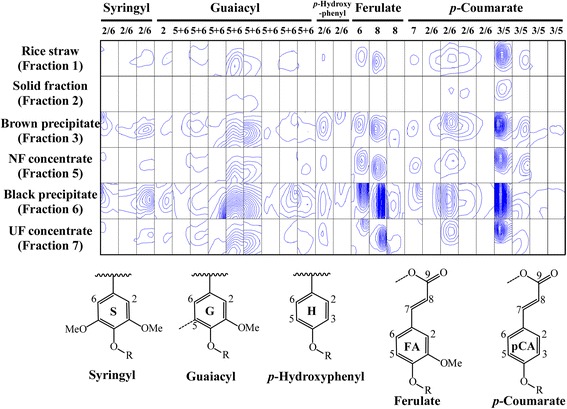
Contour plot of 2D NMR spectral regions associated with signals assigned to lignin aromatic regions. A list of chemical shifts and references for assignments are shown in Table 1

Biosynthesis of lignin involves the polymerization of three primary monolignols, *p*-coumaryl alcohol, coniferyl alcohol, and sinapyl alcohol, which generate *p*-hydroxyphenyl, guaiacyl, and syringyl subunits, respectively [3, 24, 25]. All these spectra related to syringyl and guaiacyl units decreased in the solid fraction (fraction 2), compared to rice straw (fraction 1) (Fig. 3). All these spectra increased in the brown precipitate (fraction 3). High levels of lignin aromatic regions would be released into the liquid fraction by hydrothermal pretreatment. Interestingly, these spectra increased most in the black precipitate after enzymatic hydrolysis (fraction 6), compared to rice straw, the brown precipitate, and the NF concentrate (fraction 5). These spectra decreased in the remaining UF concentrate (fraction 7). Similarly, *p*-hydroxyphenyl-related spectra were mainly increased in the brown precipitate and black precipitate and decreased in the solid fraction. As observed for syringyl and guaiacyl, *p*-coumarate and ferulate-related spectra were most increased in the black precipitate; *p*-coumarate and ferulate form ester linkages to arabinose and other aromatic constituents in the cell wall of grasses and impede bioconversion [26]. These spectra decreased in the solid fraction compared to rice straw and also decreased in the UF concentrate after removal of the black precipitate. These results suggested that lignin aromatic regions, released in the liquid fraction after hydrothermal pretreatment, were concentrated in the black precipitate after removal of hemicellulose by enzymatic hydrolysis.

### Lignin aliphatic side chain region

**Fig. 4:**
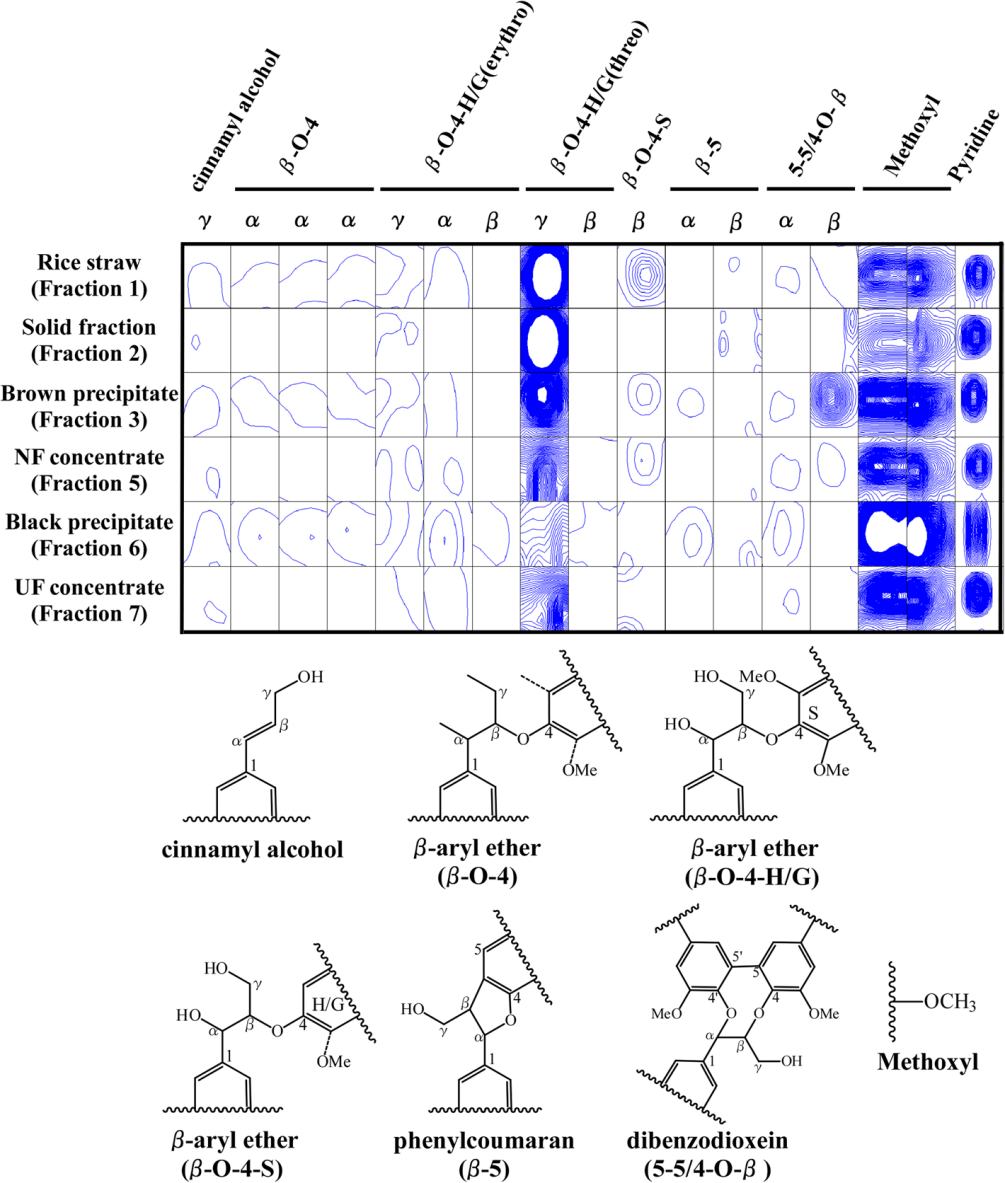
Contour plot of 2D NMR spectral regions associated with signals assigned to lignin aliphatic side chain regions. A list of chemical shifts and references for assignments are shown in Table 1

Several linkage structures (i.e., β-O-4, β-5, and 5-5/4-O-β) and cinnamyl alcohol end groups were assigned. In the linkages, a β-O-4-guaiacyl (or -*p*-hydroxyphenyl) linkage (at 60.7/3.82 ppm) was dominant in rice straw (fraction 1) (Fig. 4), which correlated with previous research that β-O-4 linkage is the major interunit of lignins [24, 27]. This β-O-4-guaiacyl linkage in rice straw remained in the solid fraction (fraction 2) and brown precipitate in the liquid fraction (fraction 3). However, this β-O-4-guaiacyl linkage was decreased in the NF concentrate (fraction 5), the black precipitate after enzymatic hydrolysis (fraction 6), and the UF concentrate (fraction 7), implying that the released lignins in the solute are degraded or depolymerized at temperatures higher than around 140–165 °C [4, 28–30]. Other β-O-4 linkages (except β-O-4-syringyl at 86.7/4.25 ppm), β-5 linkages, one 5-5/4-O-β linkage, and cinnamyl alcohol were increased in the black precipitate, compared to the NF concentrate (fraction 5). On the other hand, two methoxyl groups showed similar tendencies to aromatic regions, meaning that methoxyl groups in rice straw decreased in the solid fraction and increased in the black precipitate. These results suggested that thermally labile major β-O-4 linkages were cleaved, although minor linkages and methoxyl groups contained in aromatic regions such as syringyl and guaiacyl subunits were concentrated in the black precipitate.

### Polysaccharide regions

**Fig. 5:**
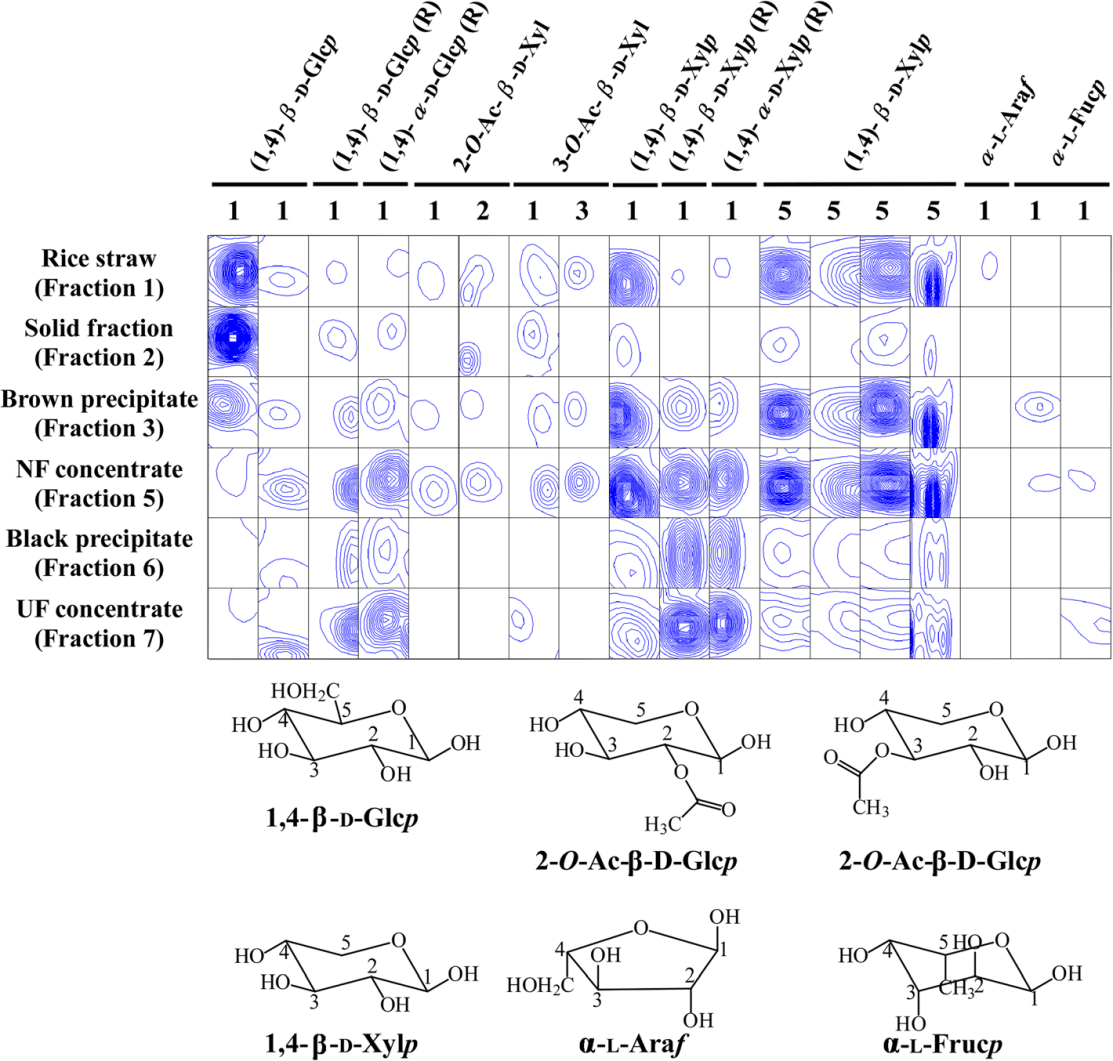
Contour plot of 2D NMR spectral regions associated with signals assigned to polysaccharide regions. A list of chemical shifts and references for assignments are shown in Table 2

(1,4)-β-D-Glc*p* (at 103.2/4.57 ppm) was the dominant cellulose in rice straw (fraction 1) (Fig. 5). Cellulose is mainly contained in the solid fraction after pretreatment [16]. As expected, the same spectrum was the major component in the solid fraction (fraction 2). This spectrum was rarely detected in the NF concentrate (fraction 5) or black precipitate (fraction 6). Spectra corresponding to another (1,4)-β-D-Glc*p*, (1,4)-β-D-Glc*p* (R) and (1,4)-α-D-Glc*p*, were increased in the NF concentrate and decreased in the black precipitate, probably due to enzymatic hydrolysis.

Reportedly, hemicelluloses are mainly solubilized in liquid fraction [16]. In accordance with previous research, five peaks corresponding to (1,4)-β-D-Xyl*p* (at 102.0/4.46, 63.5/3.46, 63.4/4.15, 63.7/4.07, and 63.3/3.34 ppm) were the major hemicellulosic components in rice straw and were increased in the brown precipitate (fraction 3) and NF concentrate. These peaks were decreased in the black precipitate, compared to the brown precipitate and NF concentrate, due to removal of the source hemicelluloses by enzymatic hydrolysis. Similarly, 2-*O*-acetylated and 3-*O*-acetylated xylose residues (2-*O*-Ac-β-D-Xyl*p* and 3-*O*-Ac-β-D-Xyl*p*) were concentrated in the NF concentrate and decreased in the black precipitate; these are reported to be abundant hemicellulose constituents of hardwoods such as aspen, birch, and beech [23, 31]. Trace of α-L-Ara*f* was observed in rice straw, and trace of α-L-Fuc*p* was observed in the brown precipitate. The decrease in α-L-Ara*f* in the solid fraction correlated with a decrease in ferulate, because ferulate is ester-linked to arabinose in grasses [26]. The intensity of two α-L-Fuc*p* peaks were also decreased in the black precipitate, compared to the NF concentrate. Therefore, polysaccharides appeared to be present at lower amounts in the black precipitate than in the solid fraction, brown precipitate, and NF concentrate of the liquid fraction.

### High lignin content in black precipitate

Lignin and carbohydrate were determined by chemical analysis of each fraction, to confirm the results obtained by solution NMR (Table 3). Lignin and carbohydrate contents accorded well with the tendencies observed in solution 2D NMR. Acid-insoluble (Klason) lignin content was highest in the black precipitate (fraction 6) and made up about half of the total mass. Soluble lignin content in the black precipitate was also twice as high as in rice straw (fraction 1). In contrast, glucan content was lowest in the black precipitate. In addition, xylan content of the black precipitate was lower than in the dried supernatant of the liquid fraction (fraction 4) and NF concentrate (fraction 5). These results supported concentration of lignin and a decrease in polysaccharides in the black precipitate. Total lignin content (acid-insoluble lignin plus soluble lignin) in the black precipitate was 65.8 %. The lignin content was higher than the value reported for NF concentrate (50 %) [13], due to the removal of polysaccharides.

**Table 3:** Lignin and carbohydrate composition of each fraction

	Fraction No.	Acid-insoluble lignin %	Soluble lignin %	Glucan %	Xylan %	Ash %	Others %
Rice straw	1	19.5	6.5	31.9	12.8	10.0	19.3
Solid fraction	2	17.2	2.5	53.6	1.1	16.5	9.1
Brown precipitate	3	29.4	8.7	13.2	13.5	4.4	30.8
Supernatant	4	17.3	9.6	8.1	19.4	0.0	45.5
NF concentrate	5	17.0	11.2	9.6	24.6	0.0	37.6
Black precipitate	6	52.9	12.9	4.0	11.9	0.1	18.3
UF concentrate	7	24.5	16.7	5.6	29.0	0.0	24.2

Based on the contents of lignin and carbohydrate (Table 3), we made a flow chart to track partitioning of lignin and carbohydrate following hydrothermal pretreatment of rice straw and the subsequent membrane separation process (Additional file 2). This flow chart started from 100 g of rice straw. As expected, lignin was separated into a solid fraction (fraction 2) and liquid fraction. Concentrated by NF after release into the supernatant of the liquid fraction (fraction 5), 52.9 % of acid-insoluble (Klason) lignin and 19.4 % of soluble lignin were recovered in the black precipitate after enzymatic hydrolysis (fraction 6). The recovered lignin does not include that lost during repeated NF concentration and dilution in the flat membrane cell used in this study. Therefore, lignin recovery can be increased further by decreasing the number of steps involved in NF concentration and dilution, for example, by recycling the concentrated liquid to the feed tank at laboratory and pilot scales [32]. On the other hand, the remaining glucan and xylan hydrolyzed in the UF permeate can be utilized for ethanol fermentation (Additional file 2) [14]. In the future, lignin should also be recovered from the solid fraction and the brown precipitate of the liquid fraction, to attain simultaneous ethanol production and recovery of lignin.

### Hypothesis for precipitation of lignin

Lignin droplets were reportedly observed on the surface of plant cell walls following hydrothermal pretreatment (at temperature ranging from 120 to 220 °C) with an acid concentration less than 4 wt% [9, 33–35]. A hydrothermal or dilute acid pretreatment, above the temperature for lignin phase transition, causes extrusion of lignin from plant cell walls and redeposit it on the surface of the cell wall during cooling. Reportedly, polysaccharides (carbohydrates) form complexes with lignin and enhance its solubility [4, 36–38]. The brown precipitate in the liquid fraction of this study (fraction 3) likely corresponds to complexes between lignin and polysaccharide after hydrothermal pretreatment (Additional file 3). Therefore, the brown precipitate contained a relatively high amount of lignin. However, the phenomenon of lignin precipitation observed during enzymatic hydrolysis of the concentrated supernatant of the liquid fraction was different from that previously observed. Conversely, hydrolysis of hemicellulose in the NF concentrate of the liquid fraction would cause release of polysaccharides from complexes with lignin and urge precipitation of lignin by decreasing the solubility of complexes. Selective precipitation of lignin in a membrane-concentrated solute would aid recovery of purified lignin.

In the future, decreasing cost is necessary for commercial usage of lignin purification process. We are currently developing a new methodology for decreasing the amount of adding hemicellulase in the purification process. In addition, to utilize recovered lignin as biofuel or precursor for carbon fibers is in investigation; however, the result in this study is a starting point for developing a commercial process for lignin recovery from lignocellulosic biomass.

## Conclusions

A precipitate with high lignin content was recovered from the supernatant of the liquid fraction of hydrothermally pretreated rice straw. The fates of lignin and polysaccharide components, during membrane separation and enzymatic hydrolysis, were evaluated in detail by 2D NMR and compositional analysis. The results of this study showed the following steps to be important in the recovery of lignin: (1) concentration of the supernatant by NF to concentrate lignins and polysaccharides (and if necessary, dilution and concentration with NF to remove acids) and (2) hydrolysis of polysaccharides by hemicellulase and recovery of the black precipitate that appeared. This black precipitate contained 65.8 % lignin, mainly aromatic components of lignin. The phenomenon observed here should aid fractionation of lignin and sugars (derived from polysaccharides) from pretreated lignocellulosic biomass, by membrane separation. Efficient lignin recovery would prevent fouling in the downstream membrane separation step and optimize ethanol fermentation from fractionated sugars, likely contributing to future utilization of recovered lignin as a value-added chemical.

## Methods

### Preparation of rice straws

Rice straw (cv. Nipponbare) was harvested in 2009 in Kansai, Hyogo, Japan. Naturally dried rice straw was shredded into pieces less than 2 mm long using WB-1 blender (Osaka Chemical Co., Ltd., Japan) fitted with a 2-mm screen. Rice straw pretreated with liquid water heated to a determined temperature (160 to 240 °C) at a pressure below 10 MPa was purchased from Mitsubishi Heavy Industries, Ltd. (Tokyo, Japan). Water was added at about a 1/10 (*w*/*v*) solid/liquid ratio [39]. After pretreatment, the liquid fraction (pH 4.4) was separated from the solid fraction by filtration through a mesh filter. The liquid fraction was stored at −20 °C until use. The brown precipitate in the liquid fraction of the hydrothermally pretreated rice straw was removed by centrifugation at 4000 rpm at room temperature for 5 min. The obtained supernatant in the liquid fraction was further separated by following membrane process.

### Membrane separation

ESNA3 polyamide NF membrane (molecular weight cut-off 150 Da) and RS50 polyvinylidene fluoride UF membrane (molecular weight cut-off 150,000 Da) were obtained from Nitto Denko Corporation (Osaka, Japan). The membranes were cut into 7.5-cm diameter circles. RS50 was soaked in 50 % (*v*/*v*) ethanol solution for 15 min, then in deionized water for 15 min, and finally soaked overnight in deionized water before use. Membrane separation was carried out at 25 °C using a flat membrane test cell (diameter 104 mm, height 147 mm, working volume 380 mL; model C40-B, Nitto Denko Corporation) on the supernatant from the liquid fraction of hydrothermally pretreated rice straw, as described previously [14]. The cell was placed on a magnetic stirrer, and the feed solution (maximum working volume 380 mL) was stirred at 400 rpm by a magnetic spin bar fitted into the cell. Pressure (2.5 and 2.8 MPa for NF and UF, respectively) was applied using nitrogen gas and regulated with a pressure control valve. The following membrane separation process (Fig. 1) was used: 6.2 times NF concentration at 2.5 MPa → [5 times dilution → NF concentration at 2.5 MPa] × 2 times (final concentration of 6.2 times) → enzymatic hydrolysis (5 g/L hemicellulase) at 37 °C for 48 h → recovery of black precipitate after centrifugation → UF permeation at 2.8 MPa. The NF concentrate was diluted with Milli-Q water. A black precipitate was recovered by centrifugation at 4000 rpm for 5 min after enzymatic hydrolysis of the NF concentrate by hemicellulase.

### Sample preparation for NMR

Pre-ground samples (150 mg) of rice straw and the solid fraction and brown precipitate in the liquid fraction of hydrothermally pretreated rice straw were extracted with water (3×) and then methanol (3×) at 50 °C for 5 min [20, 23]. These samples were lyophilized to remove water and then ball-milled for 12 h (36 × 10 min milling and 10-min cooling) using a Pulverisette 5 mill (Fritsch GmbH, Idar-Oberstein, Germany) [22]. Ball-milled samples (50 mg) were mixed with 800 μL of a 4:1 (*v*/*v*) mixture of dimethyl sulfoxide (DMSO)-*d*_6_ and pyridine-*d*_5_ and heated at 50 °C for 30 min before being centrifuged at 15,000 rpm for 5 min. The supernatant was transferred into 5-mm NMR tubes. The NF concentrate, black precipitate after enzymatic hydrolysis, and the UF concentrate were freeze-dried in an FZ-2.5 Labconco vacuum freeze-drier (Asahi Life Science Co. Ltd., Saitama, Japan), washed with deuterium oxide (Wako, Osaka, Japan) 3 times, and freeze-dried. Dried samples (50 mg) were mixed with 800 μL of a 4:1 (*v*/*v*) mixture of DMSO-*d*_6_ and pyridine-*d*_5_ and prepared in NMR tubes as described above.

### Solution 2D NMR spectroscopy

Solution NMR spectra were acquired on an AVANCE III HD-600 spectrometer (Bruker, Billerica, MA, USA), equipped with a 5-mm diameter sample probe (5 mm TCI Cryoprobe) operating at 600 MHz for ^1^H and 125 MHz for ^13^C, as described previously [22]. All of the NMR samples were maintained at 40 °C. 2D ^1^H-^13^C HSQC NMR experiments (hsqcetgp pulse program from the Bruker library) were carried out, as described previously [22, 23]. Briefly, in the ^1^H-^13^C HSQC spectra, a total of 256 complex f1 (^13^C) and 1024 complex f2 (^1^H) points were recorded with 32 scans per f1 increment. The spectral windows of the f1 and f2 dimensions were 21,124.449 Hz (140 ppm) and 8,417.509 Hz (14 ppm). The NMR data processing was performed using Topspin 3.2 software (Bruker). The chemical shifts were referred to the methyl groups of DMSO on the tetramethylsilane scale [40.03/2.583 ppm (δ_13C_/δ_1H_)]. Region of interest (ROI) was manually defined for 61 [40] using rNMR [version 1.1.8 (http://rnmr.nmrfam.wisc.edu/)] [41].

### Compositional analysis

Rice straw and the solid fraction of hydrothermally pretreated rice straw (300 mg) were hydrolyzed with 3 mL 72 % sulfuric acid according to the National Renewable Energy Laboratory (NREL) method [42]. The brown precipitate in the liquid fraction, the supernatant of liquid fraction, the NF concentrate, the black precipitate after enzymatic hydrolysis, and the UF concentrate (the moisture content 78.4, 93.2, 71.6, 42.8, and 39.6 %, respectively) were dried to 300 mg before hydrolysis. The sample was hydrolyzed at 30 °C for 60 min by rotating at 120 rpm in a PPS-25 W Chemi Station (EYELA, Tokyo, Japan). After hydrolysis, the sample was diluted with deionized water to a 4 % acid concentration and then autoclaved for 1 h at 121 °C. The residual pellet consisting of ash and acid-insoluble lignin was recovered on a Gooch type glass crucible filter (32940FNL 1G3, IWAKI, Tokyo, Japan). The resulting filtrate containing sugars and acid-soluble lignin was neutralized with CaCO_3_. The ash content was determined after burning at 575 °C for 24 h in a KDF-S70 muffle furnace (Sansyo, Tokyo, Japan). The amount of acid-soluble lignin was determined by measuring the optical density (OD) at 240 nm. Glucose and xylose concentrations in the filtrate were analyzed by a high performance liquid chromatography (HPLC) (Shimadzu, Kyoto, Japan) equipped with an Aminex HPX-87H column (Bio-Rad Laboratories, Hercules, CA, USA) operated at 65 °C using 5 mM H_2_SO_4_ as the mobile phase at a flow rate of 0.6 mL/min. The content of glucose and xylose were calibrated by hydrolysis of pure glucose and xylose (Nakalai Tesque Inc., Kyoto, Japan).

## Additional files

Additional file 1:
**2D NMR spectra of (a) solid fraction (fraction 2), (b) brown precipitate (fraction 3), (c) NF concentrate (fraction 5), and (d) UF concentrate (fraction 7).** Details of Fractions are presented in Fig. 1. Additional file 2:
**Flow chart of glucan (G), xylan (X), acid-insoluble (Klason) lignin (KL), and acid-soluble lignin (SL) in hydrothermal pretreatment of rice straw and membrane separation.** It was assumed that the process started from 100 g of rice straw. Values were observed in our experiment and calculated based on values in Table 3. Additional file 3:
**Brown precipitate (fraction 3) and black precipitate (fraction 6).** The image was taken after drying brown and black precipitates. Details of fractions are explained in Fig. 1.

## Abbreviations

2D: two-dimension
DMSO: dimethyl sulfoxide
HPLC: high performance liquid chromatography
HSQC: heteronuclear single quantum coherence
NF: nanofiltration
NMR: nuclear magnetic resonance
NREL: National Renewable Energy Laboratory
OD: optical density
ROI: region of interest
UF: ultrafiltration
