# Supplementary figures and images for: Precipitate obtained following membrane separation of hydrothermally pretreated rice straw liquid revealed by 2D NMR to have high lignin content
Source: Biotechnol Biofuels. 2015 Jun 18;8:88. doi: 10.1186/s13068-015-0273-4 (PMC4476084; doi:10.1186/s13068-015-0273-4)

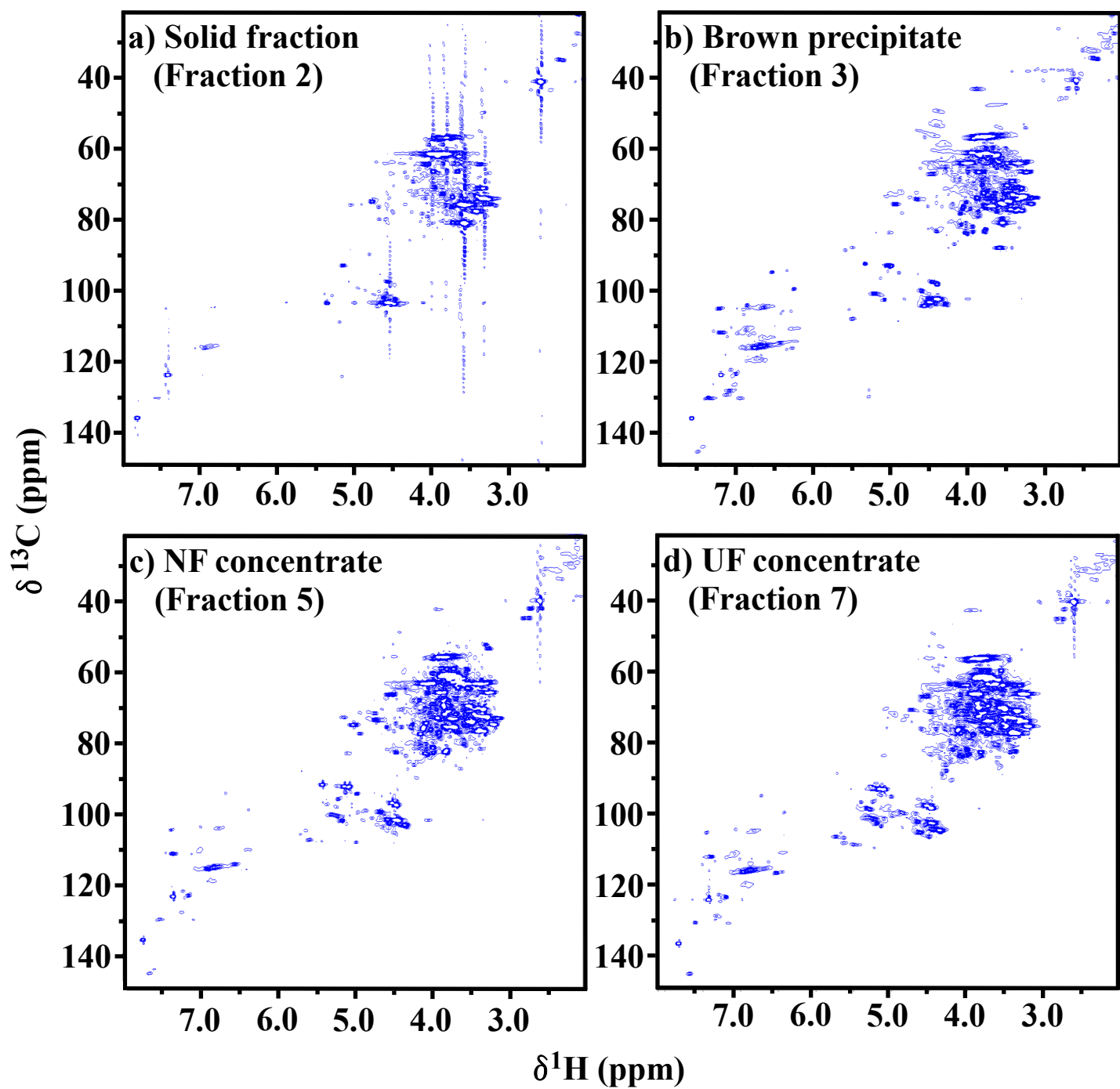

Additional file 1

Supplement: Additional file 1: — 2D NMR spectra of (a) solid fraction (fraction 2), (b) brown precipitate (fraction 3), (c) NF concentrate (fraction 5), and (d) UF concentrate (fraction 7). Details of Fractions are presented in Fig. 1. [file 13068_2015_273_MOESM1_ESM.pdf]

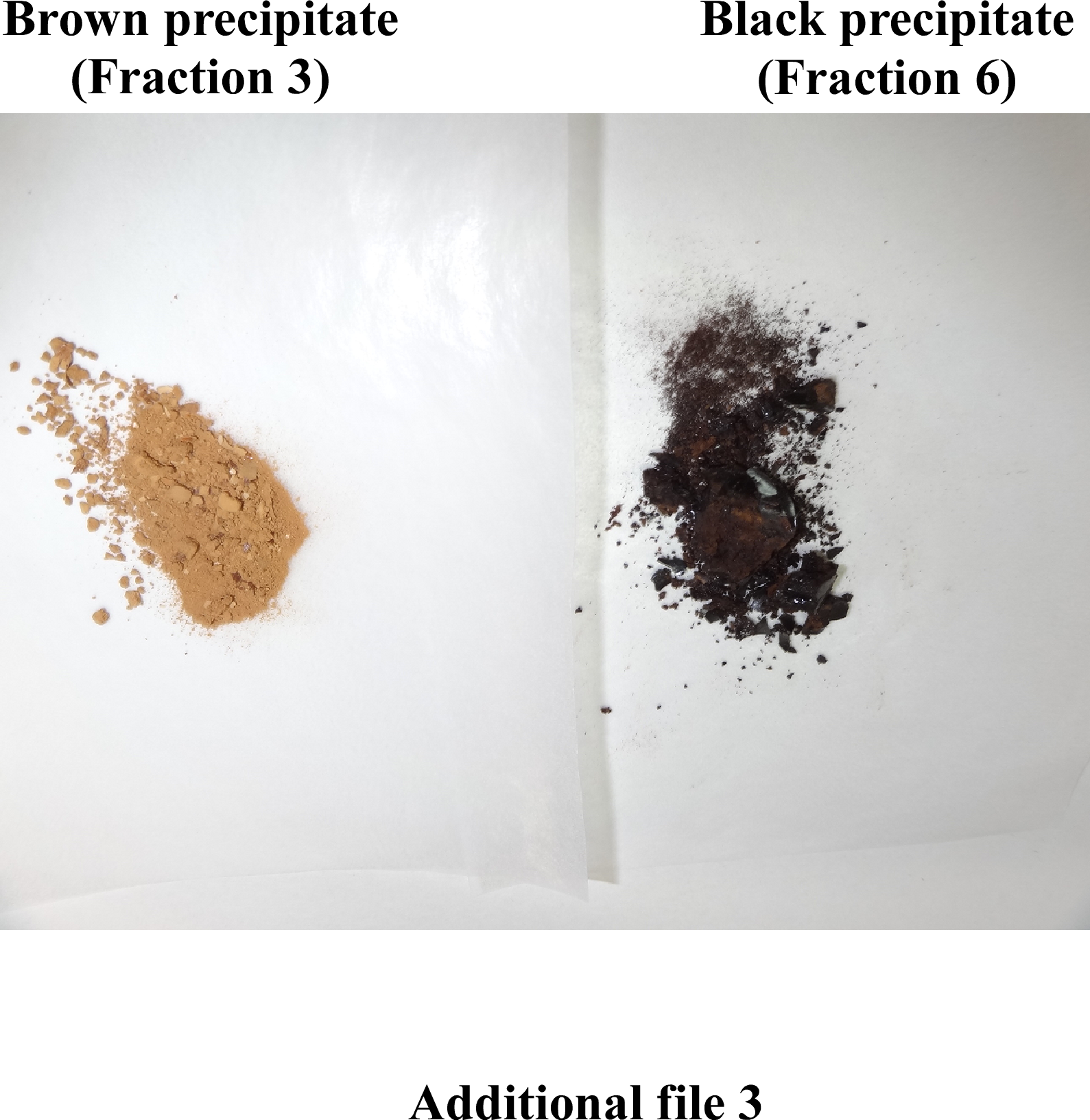

Supplement: Additional file 3: — Brown precipitate (fraction 3) and black precipitate (fraction 6). The image was taken after drying brown and black precipitates. Details of fractions are explained in Fig. 1. [file 13068_2015_273_MOESM3_ESM.tif]
